# Spatial Error Prediction and Compensation of Industrial Robots Based on Extended Joints and BO-XGBoost

**DOI:** 10.3390/s25206422

**Published:** 2025-10-17

**Authors:** Bingran Yang, Xuedong Jing

**Affiliations:** School of Intelligent Technology, Shanghai Institute of Technology, Shanghai 201418, China; jingxuedong@sit.edu.cn

**Keywords:** XGBoost, extend joint angle, industrial robots, error prediction

## Abstract

Robotic positioning accuracy is paramount in complex tasks. This accuracy is influenced by both geometric and non-geometric factors, making error prediction a significant challenge. To address this, this paper introduces two key contributions. First, we propose a novel input feature, the robot’s “extended joint angles,” which incorporates joint reversal information to better capture non-geometric errors like gear backlash. Second, we develop a high-accuracy spatial error prediction model by combining the Extreme Gradient Boosting (XGBoost) algorithm with Bayesian Optimization (BO) for hyperparameter tuning. The BO-XGBoost model establishes a direct non-linear mapping from the extended joint angles to the positioning error. Experimental results demonstrate that after compensation, the mean position error was reduced from 1.0751 mm to 0.1008 mm (a 90.62% decrease), the maximum error from 3.3884 mm to 0.4782 mm (an 85.88% decrease), and the standard deviation from 0.5383 mm to 0.0832 mm (an 84.54% decrease). A comparative analysis against Decision Tree, K-Nearest Neighbors, and Random Forest models further validates the superiority of the proposed method in reducing robot position error.

## 1. Introduction

With the rapid development of intelligent manufacturing, high-quality manufacturing technology has become a top priority for promoting economic growth, garnering further attention from the manufacturing market [1]. As a crucial carrier of intelligent manufacturing [2], industrial robots are widely used in fields such as cutting, drilling, welding, and electroplating [3]. The escalating demands of production and manufacturing processes have imposed higher precision requirements on industrial robots. However, due to assembly errors at the robot joints and errors caused by gear backlash and deformation [4], a deviation exists between the actual position of the robot’s Tool Center Point (TCP) and its theoretically programmed position. This deviation is known as the absolute positioning error. It is important to distinguish between two key performance metrics for robot positioning: absolute accuracy and repetitive accuracy. Absolute accuracy refers to the robot’s ability to move to a specific, commanded position in its workspace. In contrast, repetitive accuracy describes the robot’s ability to return to the same position consistently over multiple attempts. While many robots exhibit high repetitive accuracy, their absolute accuracy is often significantly lower due to the cumulative effect of various error sources [5]. This study focuses on predicting and compensating for the absolute positioning error to enhance the robot’s performance in high-precision tasks. Generally, the absolute positioning accuracy of many standard industrial robots is in the range of several millimeters, often lower than ±1 mm [6], making it difficult to meet the demands of high-precision tasks and thus limiting its practical application in high-end intelligent manufacturing sectors [7].

A robot’s positioning accuracy is determined by multiple factors, including its motion accuracy, structural rigidity, and controller performance [8]. After prolonged use, a robot’s structure may change due to factors like wear, accidental collisions, and positional shifts, thereby affecting its positioning accuracy. Errors arising from manufacturing and assembly of the multi-joint and multi-link structure, as well as those from long-term wear, are termed geometric errors, which account for 80% to 90% of the total error. Errors caused by factors such as temperature variations, payload, and gear backlash are termed non-geometric errors, accounting for the remaining 10% to 20% of the total error.

To solve these problems, scholars both domestically and internationally have conducted extensive research on the prediction and suppression of robot absolute positioning errors. The primary measure currently adopted is to construct an error model for the industrial robot to restore and enhance its accuracy through error compensation [9].

There are currently two main methods for improving the absolute positioning accuracy of robots: (1) online compensation and (2) offline compensation [10]. Online compensation involves real-time correction while the robot is operating, aided by various high-precision sensors. However, its operational complexity and high cost have hindered its large-scale application [11]. Offline compensation improves accuracy by establishing an error model and using a corresponding compensation system to correct the robot’s individual joint angles. It is more widely applied due to its cost-effectiveness and efficiency.

Currently, offline robot compensation can be divided into two categories:

- kinematics-based offline compensation and
- non-kinematic model-based offline compensation.

The kinematics-based calibration method can improve a robot’s positioning accuracy, but it does not account for non-geometric error factors. Consequently, the calibrated absolute positioning accuracy is often still not ideal and fails to meet higher precision requirements. Non-kinematic model-based offline compensation considers both geometric and non-geometric errors, establishing a relationship between position error and joint angles or positions without constructing a robot kinematic model [12]. This category includes methods like spatial interpolation [13] and neural network prediction compensation [14,15,16,17,18,19,20,21,22,23,24,25].

Bai Y. [26] proposed a novel model-free fuzzy interpolation method to improve the compensation accuracy for robot calibration in a 3D workspace. A comparison was made between the model-based and model-free fuzzy interpolation calibration methods. The results indicated that the model-free fuzzy interpolation method is compatible with model-based calibration and can even outperform it in terms of accuracy and complexity within a relatively compact workspace. Lightcap et al. [27] applied a flexible geometric model to a robot using the Levenberg–Marquardt algorithm to solve for the robot’s kinematic parameter errors, reducing the target robot’s mean/peak position error from 1.80/2.45 mm to 0.33/0.71 mm.

Bai, M. [28] proposed a neural network-based method to improve robot positioning accuracy. First, a genetic particle swarm algorithm was used to optimize a neural network for modeling and predicting the positioning error of an industrial robot. Subsequently, the predicted error was used to compensate for the target points in the robot’s workspace. A series of experiments under no-load and drilling scenarios validated the proposed method. The experimental results showed that the robot’s positioning error was reduced from 1.529 mm to 0.344 mm and from 1.879 mm to 0.227 mm, respectively. Cai Y. et al. [29] proposed the Kriging interpolation algorithm for calibrating off-line programmed industrial robots. This method is based on the similarity of position errors. Furthermore, it represents the position error as a deterministic drift and a residual part, considering both geometric and non-geometric errors. After calibration, the maximum spatial position error was reduced from 1.3073 mm to 0.3148 mm, and the experimental results demonstrated the algorithm’s effectiveness.

Gao T. et al. [30] compensated for non-geometric error sources in robots using an Extreme Learning Machine (ELM) neural network and validated the method’s effectiveness. Min K. et al. [31] proposed a model-free calibration method for robot position accuracy. By improving the Kriging-based error compensation method, they enhanced the performance and stability of error compensation, which could significantly reduce robot positioning errors. Du et al. [32], using a BP neural network for positioning error compensation that considered both geometric and non-geometric error factors, were able to improve the robot’s positioning accuracy by more than 80%. Similarly, Li et al. [33] also utilized an improved BP neural network to establish an error compensation model, further demonstrating the effectiveness of neural networks in this field. Hu J. et al. [34] used a GPSO-DNN optimized neural network prediction model for positioning error prediction and compensation, reducing the robot’s positioning error from 1.529 mm before compensation to 0.343 mm, an accuracy improvement of 77.57%. Beyond direct compensation methods, recent theoretical advancements have focused on a deeper understanding of the nature of positioning errors themselves. For example, studies have highlighted the importance of considering the intercorrelations among multi-point positioning errors along a trajectory, rather than treating them as independent events [35]. Zhang et al. [36] proposed a novel reliability analysis method to account for these complex statistical dependencies, providing a more accurate assessment of kinematic trajectory accuracy.

Evidently, existing research generally uses the robot’s pose as input to establish a mapping relationship with its positioning error, thereby predicting the robot’s end-effector positioning error. However, these methods only consider the pose variations in the robot’s end-effector and neglect the impact of joint reversal errors on the pose. In fact, the multi-directional repeatability error caused by joint reversal can be several times greater than the robot’s unidirectional repeatability, making it a significant factor in pose uncertainty. Nevertheless, the vast majority of current compensation methods overlook the multi-directional repeatability variations caused by joint reversal errors, which prevents further improvement in absolute positioning accuracy.

To address the aforementioned problems, this study first introduces a turning direction coefficient into the joint space to extend the robot’s joint space. Concurrently, given the superior performance of XGBoost in handling small-sample, high-dimensional, and non-linear data.

While deep learning models, such as multi-layer perceptrons, are powerful universal approximators for non-linear functions, the BO-XGBoost approach was strategically selected for this application. The key differences and advantages are

- Data Efficiency: Tree-based ensembles like XGBoost generally perform robustly on small to medium-sized tabular datasets, such as the 2000 data points collected in this study. In contrast, neural networks are often data-hungry and may require significantly more data to generalize well and avoid overfitting.
- Training Efficiency and Hyperparameter Tuning: Training XGBoost is typically faster and less computationally intensive than training a deep neural network. Furthermore, the hyperparameter space for XGBoost is more structured, making it highly suitable for efficient optimization via Bayesian methods, whereas designing and tuning NN architectures (e.g., number of layers, neurons, activation functions) can be more complex and heuristic.

A spatial error prediction model for industrial robots based on XGBoost is proposed. By incorporating Bayesian Optimization, the optimal hyperparameter combination for the XGBoost model is found efficiently and with fewer iterations. By establishing a position error prediction model, the actual position error of the robot is predicted and compensated.

Finally, the performance of the proposed BO-XGBoost model is benchmarked against other common machine learning algorithms, including Decision Tree (DT), K-Nearest Neighbors (KNN), and Random Forest (RF), to validate its superiority for this application.

The main contributions of this paper are summarized as follows:

1. A new feature set, termed “extended joint angles,” is proposed by introducing a turning direction coefficient. This allows the prediction model to account for errors arising from joint motion reversal, a factor often overlooked in existing methods.
2. An error prediction model based on Bayesian Optimized XGBoost (BO-XGBoost) is established. This data-driven approach effectively captures the complex, non-linear relationship between the robot’s configuration and its positioning error without requiring a complete kinematic model.

The remainder of this paper is organized as follows.

- Section 2 details the robot error analysis, the principles of the XGBoost algorithm, and the construction of the optimal model using Bayesian Optimization.
- Section 3 describes the experimental setup, including the Leica AT960 laser tracker system, and the data processing procedures.
- Section 4 presents and discusses the experimental results in detail. This section first demonstrates the significant error reduction achieved by the proposed method. Subsequently, a crucial ablation study is conducted, which isolates and quantifies the specific accuracy improvement obtained from the introduction of the novel “extended joint angles” feature. Following this, a comparative analysis is performed to benchmark the BO-XGBoost model against the Decision Tree, K-Nearest Neighbors, and Random Forest models, thereby validating its superior performance. Finally, the practical effectiveness of the model in a dynamic scenario is verified through a continuous 180 mm × 180 mm square path tracking experiment.
- Section 5 concludes the paper, summarizing the findings, acknowledging the study’s limitations, and suggesting directions for future research.

## 2. Principles and Methods

### 2.1. Error Analysis

**Figure 1:**
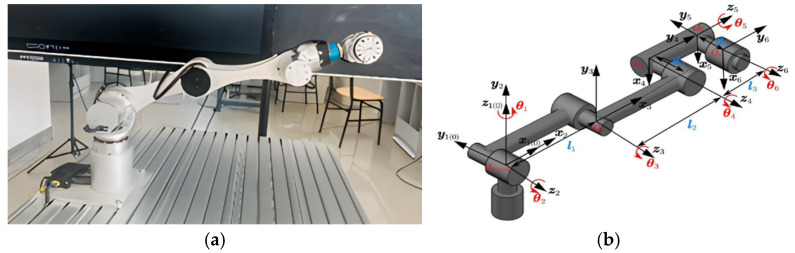
Schematic diagram of the joint coordinate system of DrCbot-6 industrial robot. (**a**) The physical setup of the industrial robot; (**b**) Schematic diagram of the joint coordinate system.

The DrCbot-6 industrial robot used in this paper is a 6-degree-of-freedom serial robot. The schematic diagram of its mechanical structure is shown in Figure 1a, and the schematic diagram of the joint coordinate system is shown in Figure 1b.

The kinematic model of the six-axis robot is established based on the Modified Denavit-Hartenberg (MD-H) parameter method. The transformation matrix from link coordinate system to link coordinate system is defined by Equation (1), with its expanded homogeneous transformation matrix shown in Equation (2) [37]:

(1) Tii−1=RotXi−1,αi−1·TransXi−1,ai−1·RotZi,θi·TransZi,di

(2) Tii−1=cosθi−sinθi0αi−1sinθicosαi−1cosθicosαi−1−sinαi−1−disinαi−1sinθisinαi−1cosθisinαi−1cosαi−1dicosαi−10001

In Equation (1): Rot is the rotation transformation matrix about the axis; Trans is the translation transformation matrix along the axis; $\alpha_{i-1}$ and $a_{i-1}$ are the link twist angle and link length of link {i−1}, respectively; $\theta_{i}$ and $d_{i}$ are the joint angle and link offset of link {i}, respectively. Based on the joint coordinate system established in Figure 1, the MD-H model parameters for the industrial robot to be calibrated are presented in Table 1.

The transformation matrix T60 of the robot kinematic model can be expressed as [37]:

(3) T60=T10T21T32T43T54T=P65

where $T_{n}$ is the homogeneous transformation matrix of the robot end-effector relative to the base coordinate system, P is the position vector of the robot end-effector. The theoretical position matrix $P_{n}$ can be expressed as [37]

(4) $$P_{e}=\left[\begin{array}{c} x_{n}\\ y_{n}\\ z_{n} \end{array}\right]$$

The actual position matrix $P_{r}$ can be expressed as [37]

(5) $$P_{r}=\left[\begin{array}{c} x_{r}\\ y_{r}\\ z_{r} \end{array}\right]$$

The position error vector ∆p of an industrial robot is the vector difference between the actual position vector and the theoretical position vector.

(6) $$∆p=p_{e}-p_{n}=\left[\begin{array}{c} x_{r}-x_{n}\\ y_{r}-y_{n}\\ z_{r}-z_{n} \end{array}\right]=\left[\begin{array}{c} ∆x\\ ∆y\\ ∆z \end{array}\right]$$

In practical applications, the primary concern is typically the absolute magnitude of the position error, rather than its components along the individual axes. The overall error $E_{p}\;$ can be obtained using the Euclidean norm:

(7) $$E_{p}=∥∆p∥=\sqrt{{∆x}^{2}+{∆y}^{2}+{∆z}^{2}}$$

Based on Equation (1), the equation for calculating the position P of the robot end-effector in the robot base frame can be expressed as

(8) P=T60=fd,θ,α,a

where d,θ,α, a are robot parameters.

The differential equation of Equation (8) can be expressed as

(9) $$E_{p}=\frac{\partial f}{\partial d}∆d+\frac{\partial f}{\partial \theta }∆\theta +\frac{\partial f}{\partial \alpha }∆\alpha +\frac{\partial f}{\partial a}∆a$$

where ∆d,∆θ,∆α,∆a are robot parameters.

For the robot, the error parameters ∆d,∆α,∆a are all constants; therefore, Equation (9) can be replaced by Equation (10).

(10) $$E_{P}=g\left(\theta \right)$$

In the equation, $g\left(\theta \right)$ is the representation of the custom position error function, expressed as a function $g\left(\theta \right)$ of θ.

From Equation (10), it can be seen that the position error $E_{p}$ is a function of the joint angles θ; therefore, the joint angles can be considered to be related to the position error.

While Equation (9) provides a differential kinematic framework, directly modeling all error sources, especially non-geometric ones like gear backlash, within this framework is exceedingly complex and often leads to intractable non-linear equations. Therefore, instead of pursuing a purely analytical model, this study adopts a data-driven, non-kinematic approach. We use the kinematic relationship established in Equation (10) as a basis to select model inputs. The core idea is to build a machine learning model that directly maps the robot’s state (including factors that influence errors) to the resulting position error.

Due to gear backlash in the robot’s joint motors, the actual rotation angle deviates from the theoretical value when the direction of joint movement changes [38]. Research indicates that this error is a function of the theoretical joint angle and the direction of motion, and that the absolute magnitude of the error is equal in both forward and reverse movements [39]. Therefore, the actual joint angle $\tilde{\theta_{i}}$ can be expressed as a function of the theoretical angle $\theta_{i}$ and the direction of motion [40], as shown in Equations (11) and (12).

(11) $${\tilde{\theta }}_{i}=\theta_{i}+\lambda_{i}b_{i}\left(\theta_{i}\right)$$

(12) $$\lambda_{i}=\left\{\begin{array}{c} 1,\;\;\;\;\;\;forword\;motion\\ -1,\;\;\;\;\;\;backword\;motion \end{array}\right.$$

In robotic joints driven by gears, a phenomenon known as backlash occurs. When a joint reverses its direction of rotation, there is a small amount of play or clearance before the gear teeth re-engage. This results in a position error that is dependent on the direction of the final approach to a target angle. The coefficient λ is designed to explicitly provide this directional information to the prediction model. For each joint i, we define $\lambda_{i}=+1$ if the joint rotates in its positive direction to reach the target angle, and $\lambda_{i}=-1$ if it rotates in the negative direction. By incorporating this binary coefficient, the feature space is “extended” to allow the model to learn two different error patterns for the same target angle, depending on how it was approached. Where $\tilde{\theta_{i}}$ is the actual joint angle after considering the change in joint turning direction; $\theta_{i}$ is the theoretical joint angle; $\lambda_{i}$ is the turning direction coefficient; and $b_{i}\left(\theta_{i}\right)$ is the gear backlash error.

Therefore, for a robot with n joints, its joint space can be expanded from $\left[\theta_{1},\theta_{2},\cdots ,\theta_{n}\right]$ to $\left[\theta_{1},\theta_{2},\cdots ,\theta_{n},\lambda_{1}{,\lambda }_{2},\cdots ,\lambda_{n}\right]$. This is done so that the model can reflect the effect of errors arising from the direction of joint rotation on the robot’s final absolute positioning accuracy.

### 2.2. XGBoost

Gradient Boosting Decision Tree (GBDT), proposed by Friedman in 2001, is a boosting algorithm. While the standard gradient boosting algorithm only utilizes first-order derivatives in its objective function, XGBoost is an improvement upon it. The core idea of XGBoost is to combine multiple weak learners into a single strong classifier. It extends the loss function by introducing regularization and a second-order Taylor series expansion, which effectively controls model complexity, allows the model to converge rapidly [41,42], and reduces the risk of overfitting [43]. XGBoost is significantly superior to the traditional gradient boosting algorithm in terms of both prediction accuracy and training speed.

The regularized objective function ${Obj}^{\left(t\right)}$ for XGBoost is given as

(13) $${Obj}^{\left(t\right)}=\sum_{i=1}^{n}l\left(y_{i},{{\hat{y}}_{i}}^{\left(t\right)}\right)+\sum_{k=1}^{K}\Omega \left(f_{k}\right)$$

where K is the number of classification and regression trees, and $f_{k}$ is the model for the K st tree.

$l\left(y_{i},{\hat{y}}_{i}\right)$ is the loss function, which determines the empirical risk, and its definition is as shown in the equation:

(14) $$l\left(y_{i},{\hat{y}}_{i}\right)={\left({\hat{y}}_{i}-y_{i}\right)}^{2}$$

where ${\hat{y}}_{i}$ is the predicted value, and $y_{i}$ is the true value.

$\Omega \left(f_{k}\right)$ is the structural risk, which adds the number of leaf nodes as a penalty term to limit model complexity and uses $L_{2}$ regularization to avoid overfitting; its definition is shown in equation:

(15) $$\Omega \left(f\right)=\gamma T+\frac{1}{2}\lambda \sum_{j=1}^{T}\omega_{j}^{2}$$

where T is the number of leaf nodes; γ is the minimum loss required to further partition a leaf node; λ is the regularization parameter, primarily used for scaling the penalty; and $w_{j}$ is the weight of the leaf node.

For the t-th iteration, the objective function $L^{\left(t\right)}$ can be written as

(16) $$L^{\left(t\right)}=\sum_{i=1}^{n}l\left(y_{i},{{\hat{y}}_{i}}^{\left(t-1\right)}+f_{t}\left(x_{i}\right)\right)+\Omega \left(f_{t}\right)$$

where $f_{t}\left(x_{i}\right)$ is the predicted value of the t-th tree for the i-th sample.

The objective function can be rapidly optimized through a second-order expansion:

(17) $$L^{\left(t\right)}≅\sum_{i=1}^{n}\left[l\left(y_{i},{\hat{y}}^{\left(t-1\right)}\right)+g_{i}f_{t}\left(x_{i}\right)+\frac{1}{2}h_{i}f_{t}^{2}\left(x_{i}\right)\right]+\Omega \left(f_{t}\right)$$

where $g_{i}$ and $h_{i}$ are the first-step and second-step statistics of the loss function, respectively. After removing the constant terms, the sample set of the j-th leaf is defined as $I_{j}=\left\{i|q\left(x_{i}\right)=j\right\}$, where $q\left(x_{i}\right)$ is the prediction function. The objective function is

(18) $$L^{\left(t\right)}=\sum_{j=1}^{T}\left[\left(\sum_{i\in I_{j}}g_{i}\right)\omega_{j}+\frac{1}{2}\left(\sum_{i\in I_{j}}h_{i}+\lambda \right)\omega_{j}^{2}\right]+rT$$

where $\omega_{j}$ is the weight of leaf j. For a fixed structure, the optimal solution $\omega_{j}^{*}$ can be calculated as follows:

(19) $$\omega_{j}^{*}=-\frac{\sum_{i\epsilon I_{j}}g_{i}}{\sum_{i\epsilon I_{j}}h_{i}+\lambda }$$

and the corresponding value is calculated as follows:

(20) $$L^{\left(t\right)}=-\frac{1}{2}\sum_{j=1}^{T}\frac{{\left(\sum_{i\epsilon I_{j}}g_{i}\right)}^{2}}{\sum_{i\epsilon I_{j}}h_{i}+\lambda }+rT$$

The above equation can serve as a scoring function to measure the quality of a tree structure; a smaller value indicates a better structure. However, enumerating all possible tree structures to find the optimal solution is difficult and computationally infeasible.

Therefore, XGBoost employs a greedy algorithm to approximately find the optimal tree structure. The basic idea is to start from a root node (a tree of depth 0) containing all samples and perform splits level by level. For each leaf node, the algorithm attempts to iterate through all possible split points for every feature and calculates the gain brought by each split.

The equation for the split gain is as follows:

(21) $$Gain=\frac{1}{2}\left[\frac{{\left(\sum_{i\in I_{L}}g_{i}\right)}^{2}}{\sum_{i\in I_{L}}h_{i}+\lambda }+\frac{{\left(\sum_{i\in I_{R}}g_{i}\right)}^{2}}{\sum_{i\in I_{R}}h_{i}+\lambda }-\frac{{\left(\sum_{i\in I}g_{i}\right)}^{2}}{\sum_{i\in I}h_{i}+\lambda }\right]-\gamma$$

where $I_{L}$ and $I_{R}$ are the sets of sample instances that go into the left and right child nodes after the split, respectively, and $I=I_{L}\cup I_{R}$ is the set of sample instances for the node before the split. The first term is the score of the left subtree after the split, the second term is the score of the right subtree, the third term is the score of the node before the split, and γ is the extra penalty for introducing a new leaf node.

The algorithm will select the feature and its corresponding split point that maximize the gain as the optimal split point for the current step. If the calculated maximum gain is less than 0, it means that the benefit from this split is not sufficient to cover the cost (γ) of adding a new leaf node. In that case, the node will stop splitting and become a leaf node. By continuously repeating this process, XGBoost iteratively constructs the entire decision tree.

**Figure 2:**
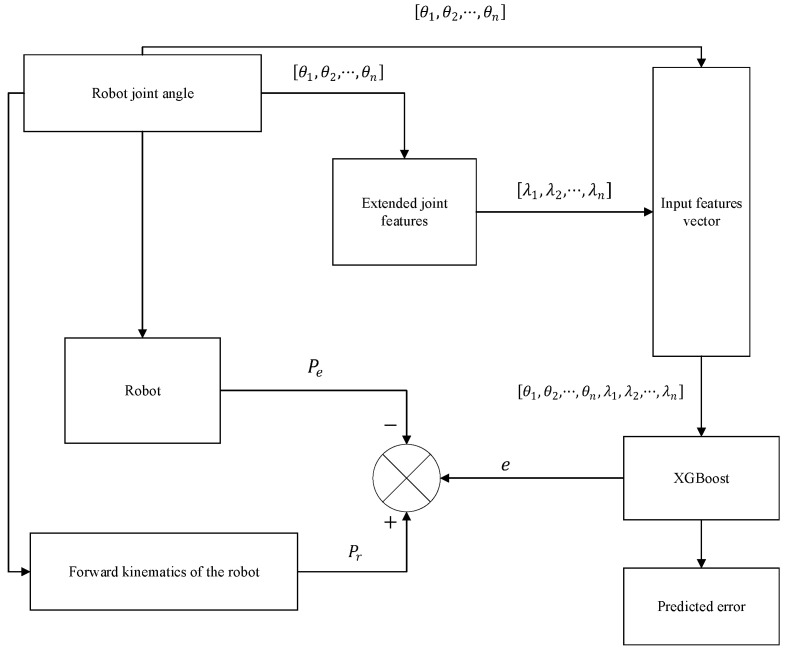
Flowchart of the XGBoost model training process.

The training process of XGBoost is shown in Figure 2:

The model is trained using the theoretical joint angles $\left(\theta_{1}\sim \theta_{6}\right)$ combined with the turning direction coefficients $\left(\lambda_{1}\sim \lambda_{6}\right)$ as the input features. This complete 12-dimensional vector represents the ‘extended joint angles’ and is used to predict the measured position error e, which serves as the target label.

### 2.3. Optimal Model Construction

XGBoost is an efficient and flexible gradient boosting framework in the field of machine learning, but its performance is highly dependent on its hyperparameter settings [44]. Traditional Grid Search and Random Search are commonly used hyperparameter tuning methods in machine learning practice [45,46]. Grid Search determines a suitable optimal combination by trying all possible hyperparameter combinations within the search space. However, Grid Search is susceptible to the curse of dimensionality, meaning the computational load during the optimization process grows exponentially with the number of hyperparameters, making it infeasible for an XGBoost model with numerous hyperparameters. Random Search seeks the optimal hyperparameter combination by searching through random combinations; however, due to its completely random process, there is no guarantee of finding the globally optimal hyperparameter combination.

**Figure 3:**
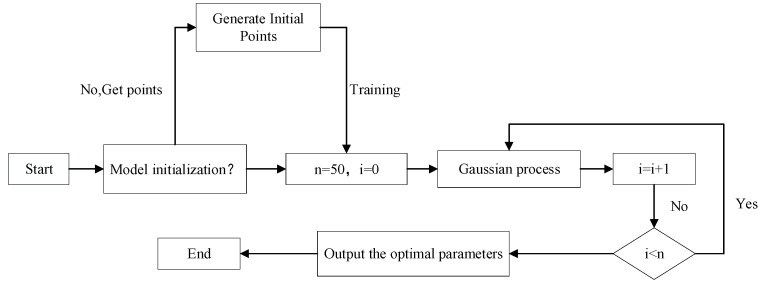
Bayesian optimization flowchart.

The Bayesian Optimization method [47,48] is particularly suitable for improving model performance, as it requires fewer iterations and offers higher optimization efficiency than Grid Search and Random Search. Its optimization process is illustrated in Figure 3, and this process follows the iterative steps outlined below:

1. Objective Setting

First, we define the following XGBoost parameters: n_estimators, max_depth, learning_rate, subsample and reg_lambda as an objective function $f\left(x\right)$. This function maps the hyperparameters x to a scalar performance metric, In this study, the objective function f(x)  to be maximized is the negative Root Mean Squared Error (RMSE) evaluated through 5-fold cross-validation on the training dataset. Maximizing the negative RMSE is equivalent to minimizing the RMSE, which guides the search towards hyperparameter combinations that yield better predictive accuracy, and the goal is to identify the vector $x^{*}$ that maximizes this function:

(22) $$x^{*}=argmaxf\left(x\right)$$

2. Prior Probability Distribution

Let each hyperparameter $f\left(x\right)$ of XGBoost follow a prior probability distribution.

(23) $$f\left(x\right)\sim GP(m\left(x\right),k\left(x,x^{\prime }\right))$$

where $m\left(x\right)$ is the mean function, and $k\left(x,x^{\prime }\right)$ is the covariance kernel function, which is used to measure the correlation between points.

3. Initial stage

In the initial stage of optimization, we randomly sample $n_{0}$ set of hyperparameter combinations and evaluate the true value of the corresponding objective function. This forms the initial observation dataset $D_{n}={\left\{\left(x_{i},y_{i}\right)\right\}}_{i=1}^{n}$, where $y_{i}=f\left(x_{i}\right)$.

4. Optimization Process

This is the core of Bayesian Optimization. Based on the existing observation data $D_{i}$, we update the posterior distribution $P\left(f|D_{t}\right)$ of the Gaussian process. Subsequently, an “acquisition function, α(x)” is used to decide the next most promising point to evaluate. The commonly used “Expected Improvement, EI” acquisition function aims to maximize the expected improvement over the current best value $f\left(x^{+}\right)$.

(24) $$x_{t+1}={argmax}_{x\in X}\alpha_{EI}\left(x\right)={argmax}_{x\epsilon X}E\left[max\left(f\left(x\right)-f\left(x^{+}\right),0\right)\right]$$

This function effectively balances exploration and exploitation to determine a potential candidate hyperparameter combination. This combination is then used to train a new XGBoost model, and its performance is evaluated again.

5. Iterative Update

Evaluate the true objective function $f\left(x_{t+1}\right)$ at the new point $x_{t+1}$ determined by the acquisition function to obtain the new observation $y_{t+1}$. Subsequently, this new data point $\left(x_{t+1},y_{t+1}\right)$ is added to the observation set to form $D_{t+1}=D_{t}\cup \left\{\left(x_{t+1},y_{t+1}\right)\right\}$. This process provides a more accurate posterior distribution for the next iteration, thereby guiding a more precise search.

6. Termination Condition

The Bayesian optimization process continues until a preset number of iterations is reached or the model’s performance meets the convergence criterion.

The hyperparameters optimized in this study and their results are presented in Table 2; all other parameters were set to their default values.

A single set of optimal hyperparameters, as determined by the Bayesian Optimization process Table 2, was used to configure three separate XGBoost models. These models were trained independently to predict each component of the three-dimensional position error vector  (Δx, Δy, Δz). Each model takes the 12-dimensional extended joint angle vector as input and is specialized in predicting one error component (Δx,Δy,Δz).

**Figure 4:**
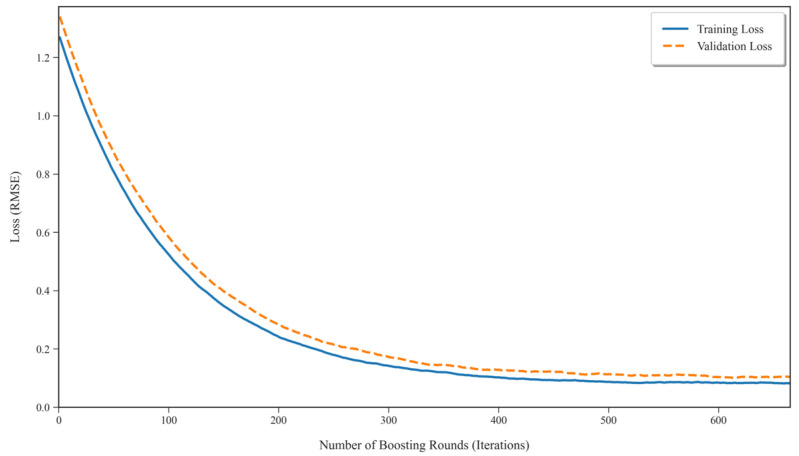
Training and validation loss curves for the BO-XGBoost model.

To visualize the training process and verify model convergence, the training and validation loss curves were plotted against the number of boosting iterations, as shown in Figure 4. The loss function, typically Root Mean Squared Error (RMSE) for regression tasks, steadily decreases for both the training and validation sets and then plateaus, indicating that the model has converged effectively without significant overfitting. This visualization supports the selection of 665 estimators as a suitable value, balancing model complexity and generalization performance.

### 2.4. Error Compensation Procedure

**Figure 5:**
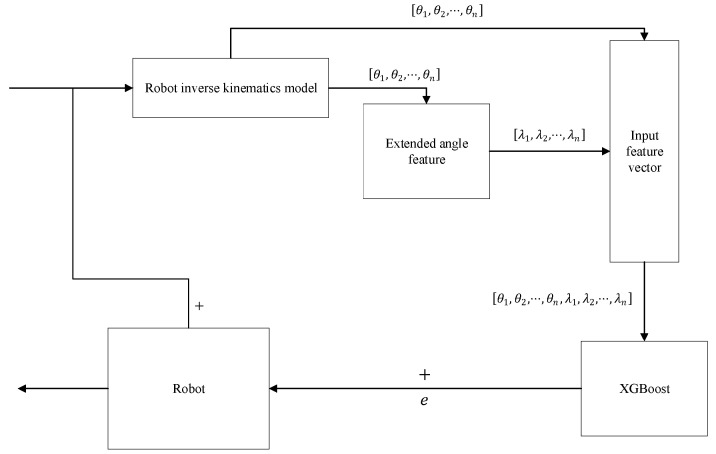
Flowchart of the error compensation process.

Once the BO-XGBoost models are trained, they can be deployed to compensate for position errors in real-time or offline. The compensation process follows the flowchart shown in Figure 5. For a given desired target position, the corresponding joint angles are calculated via inverse kinematics. These angles, combined with the direction of motion for each joint, form the extended joint angle vector. This vector is fed into the three trained XGBoost models to predict the error components $\;(\Delta \hat{x},\Delta \hat{y},\Delta \hat{z})$. The predicted error is then subtracted from the original target position to generate a compensated target, which is sent to the robot controller. This effectively pre-corrects the command to counteract the robot’s inherent positioning error.

## 3. Experiments

### 3.1. Calibration System

**Figure 6:**
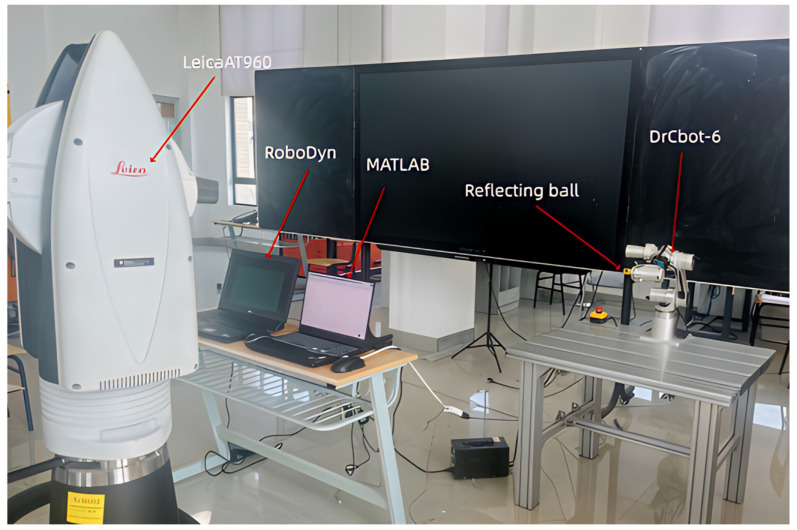
Industrial robot calibration system.

Figure 6 Industrial robot calibration system shows the industrial robot calibration system built for this study. The system utilizes a Leica AT960 laser tracker (Leica Geosystems, Heerbrugg, Switzerland) and a reflector ball as the tracking measurement system to calibrate the DrCbot-6 industrial robot. The measurement uncertainty of the Leica AT960 laser tracker is $\pm \left(15\;\mu m+6\;\mu m/m\right)$. The DrCbot-6 industrial robot has a rated payload of 3 kg and a maximum payload of 5 kg. It is acknowledged that the robot’s internal angular sensors (encoders) are a source of measurement error that contributes to the final positioning inaccuracy. While the robot’s motion is commanded by theoretical joint angles sent from MATLAB (version 2023a), the execution of these commands relies on a closed-loop feedback system governed by these internal sensors. A key advantage of the proposed data-driven, non-kinematic compensation method is that it does not require explicit modeling of individual error sources like encoder inaccuracies. By directly learning the mapping from the commanded joint angles to the measured end-effector error, the XGBoost model implicitly captures the cumulative effect of all systematic error sources, including those originating from the angular sensors.

The measurement method using the laser tracker is as follows:

(1) Reflector Installation: A reflector ball is mounted on the robot’s end-effector tool. The reflector ball is capable of reflecting the laser beam, which, in conjunction with the laser tracker, allows for real-time data acquisition of the reflector ball’s center position.

(2) Laser Tracker Installation and Configuration: The laser tracker is configured to emit a laser beam and track the reflector ball’s position, collecting data in a prescribed manner. Concurrently, the reflector ball model, data acquisition mode, and other settings must be configured in the software.

(3) Point and Path Design: Points are defined within the robot’s workspace according to the desired plan. MATLAB software is used to control the theoretical positions along the industrial robot’s path, while the laser tracker captures the actual positions of these points for subsequent calculations. It is crucial to ensure that all points during the robot’s movement can be properly measured, as the reflector ball has a limited line-of-sight range, to prevent the beam from being lost.

(4) End-Effector Position Measurement: As the robot operates, the laser tracker continuously tracks the reflector ball’s position. By measuring the reflection angles of the laser beam, the spatial position of the reflector ball relative to the laser tracker can be calculated. The accompanying measurement and analysis software used is RoboDyn (version 1.2.9033.17186), which provides functionalities such as robot modeling, coordinate system creation, and calibration, and can independently calculate the actual position of the robot’s end-effector.

### 3.2. Data Preprocessing

**Figure 7:**
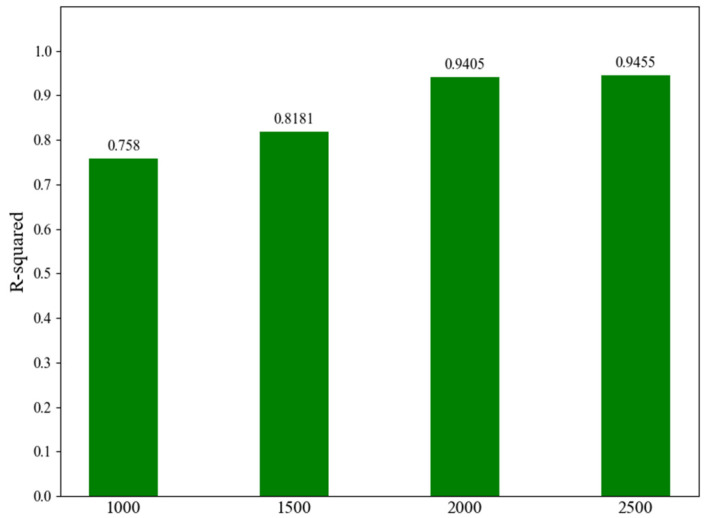
Analysis of model performance (R-squared) versus dataset.

A comprehensive dataset was collected to evaluate and compensate for the robot’s absolute positioning accuracy. To determine the optimal size of this dataset, The results of this analysis are presented in Figure 7. As illustrated, the model’s performance, measured by the R-squared score, improves substantially as the dataset size increases from 1000 to 2000 points. Specifically, the R-squared score jumps from 0.758 to 0.9405, indicating that the model was significantly under-resourced with fewer than 2000 data points.

However, a further increase in the dataset size from 2000 to 2500 points yielded only a marginal improvement in the R-squared score, from 0.9405 to 0.9455. This represents a performance gain of only 0.5%, which is negligible considering the additional 25% effort required for data collection. Therefore, a dataset of 2000 points was identified as the optimal trade-off, providing near-maximum model performance without the prohibitive cost of further data acquisition.

**Figure 8:**
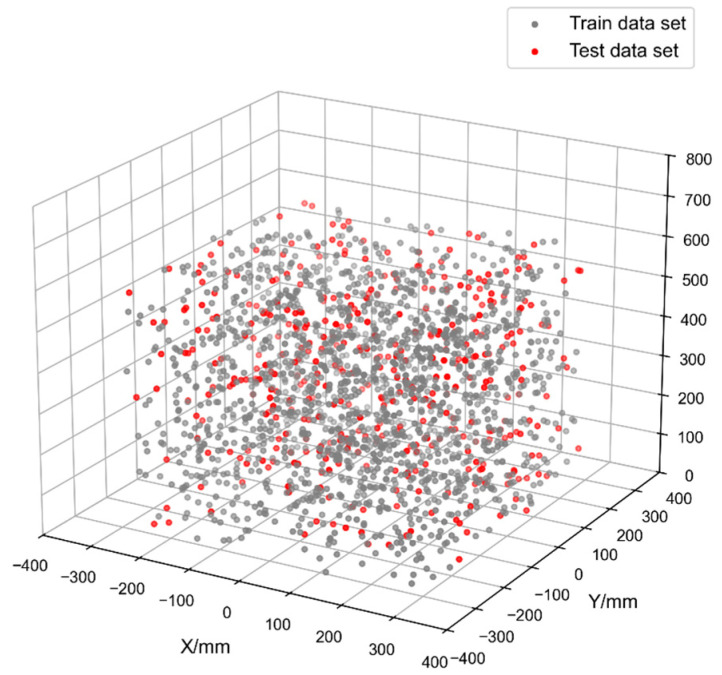
Spatial Distribution of Position Measurement Points for Industrial Robots.

Accordingly, a comprehensive dataset comprising these 2000 reachable positions was generated using MATLAB software. The points were randomly distributed within an 800 mm cubic volume located in the robot’s primary workspace. The resulting spatial distribution of the collected data points is shown in Figure 8.

Due to the heterogeneous scales of the variables, where input features include joint angles $x_{i}=\left(\theta_{1i},\theta_{2i},\theta_{3i},\theta_{4i},\theta_{5i},\theta_{6i},\lambda_{1i},\lambda_{2i},\lambda_{3i},\lambda_{4i},\lambda_{5i},\lambda_{6i}\right)$ and the target values are position errors $\;\left(dx,dy,dz\right)$, data normalization was employed. This preprocessing step prevents features with larger numerical ranges from disproportionately influencing the model’s training process. Specifically, Min-Max scaling was applied to transform both the input features and the target values into the uniform interval of [−1,1], according to Equation (25):

(25) $$u^{\prime }=\frac{\left(v_{max}-v_{min}\right)\times \left(u-u_{min}\right)}{u_{max}-u_{min}}+v_{min}$$

where $u^{\prime }$ is the data to be normalized; $u_{max}$ and $u_{min}$ represent the maximum and minimum values of u, respectively, with the target interval $\left[v_{min},v_{max}\right]$ set to $\left[-1,1\right]$, mapping the data to within interval $\left[-1,1\right]$. Although tree-based models such as XGBoost are generally less sensitive to feature scaling, this normalization is adopted as a robust practice to enhance numerical stability. The [−1,1] interval was chosen as it is a standard convention that also centers the data around zero.

Following normalization, the dataset was randomly divided into a training set, comprising 80% of the data, and a test set for the remaining 20%.

From the analysis of the overall position error of the dataset, the mean error is 1.0751 mm, the maximum error is 3.3884 mm, and the standard deviation is 0.5383 mm.

## 4. Result

It is important to clarify the nature of the compensation applied in this study. Unlike kinematics-based calibration methods that identify and correct specific geometric parameters (e.g., DH parameter errors), the proposed XGBoost-based approach is non-kinematic and model-free. It does not compute or visualize deviations in a DH table or backlash values. Instead, it creates a predictive model that directly maps a given robot configuration (extended joint angles) to the expected spatial positioning error  (Δx,Δy,Δz). The compensation is then applied by adjusting the target coordinates with the negative of the predicted error. The following sections present the performance of this data-driven compensation by comparing the robot’s positioning error before and after this corrective process.

### 4.1. Experimental Analysis of BO-XGBoost

**Figure 9:**
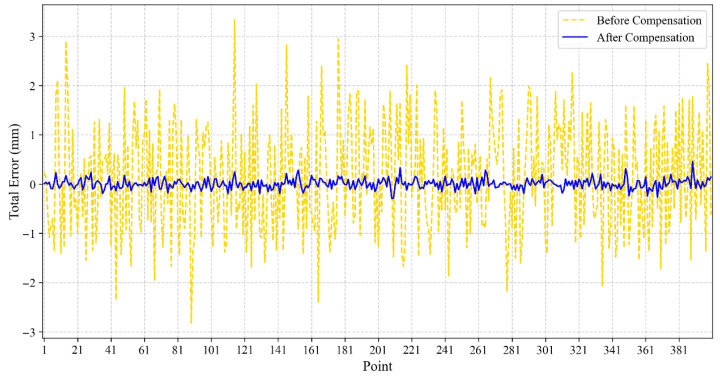
Comparison of positional errors along the *x*-axis before and after BO-XGBoost compensation.

The authors, using simulations, compared the position errors before and after compensation using the BO-XGBoost method proposed in this paper. The position errors of 400 sample points along the *x*-axis, before and after compensation, are plotted as shown in Figure 9.

**Figure 10:**
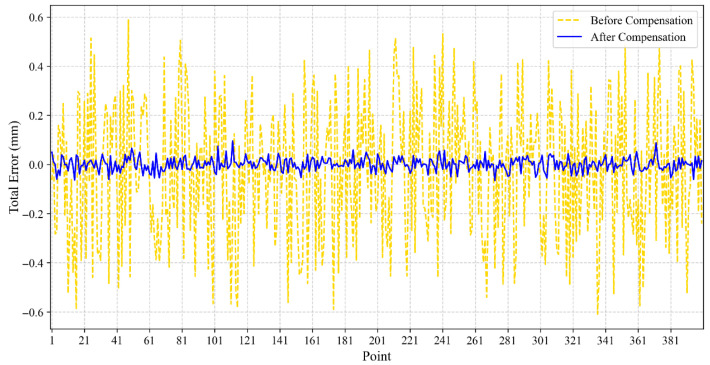
Comparison of positional errors along the *y*-axis before and after BO-XGBoost compensation.

Figure 9 illustrates that the position error before compensation fluctuates within a large range, whereas the compensated error (blue line) is consistently close to zero. while the position error is significantly reduced after compensation. The position errors for the 400 sample points in the Y-direction, before and after compensation, are shown in Figure 10:

Similarly, Figure 10 shows a significant reduction in both the offset and amplitude of the *y*-axis error after compensation, with the mean error shifting from approximately 0.5 mm to near 0 mm, decreasing from 0.5 mm to approximately 0 mm, and the amplitude of the error oscillation has also diminished.

**Figure 11:**
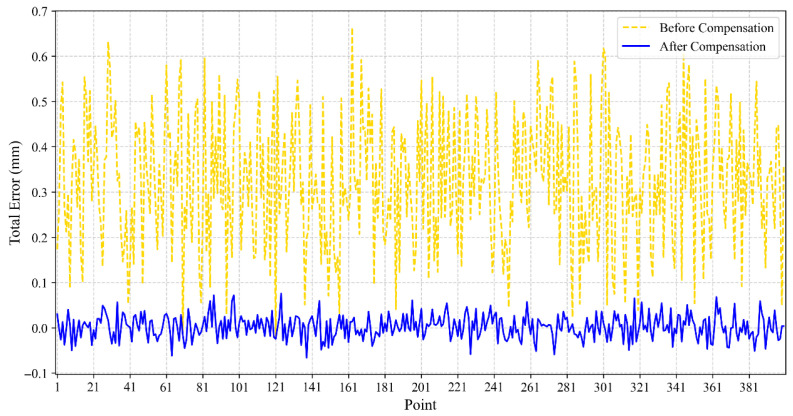
Comparison of positional errors along the *z*-axis before and after BO-XGBoost compensation.

The position errors of 400 sample points in the Z-direction, before and after compensation, are shown in Figure 11.

As can be seen from the figure, after compensation, the overall offset of the robot’s position error in the Z-direction is significantly reduced.

**Figure 12:**
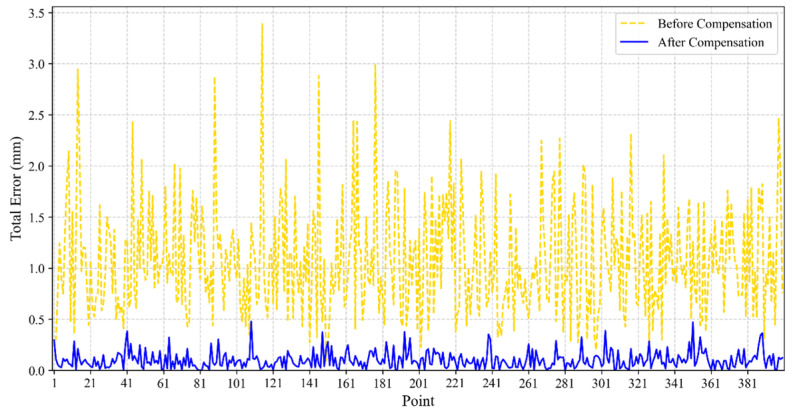
Comparison of total Euclidean position errors for 400 test points before (yellow) and after (blue) compensation.

Furthermore, to more clearly show the overall position error compensation results, the authors have plotted a comparison of the total position errors before and after compensation, as shown in Figure 12.

It can be seen that the robot’s pose error is effectively suppressed. The total position error was reduced from a maximum of 3.3884 mm to 0.4782 mm, and the mean position error was reduced from 1.0751 mm to 0.1008 mm, representing decreases of 85.88% and 90.62%, respectively. The experimental results indicate that the error prediction and compensation method proposed in this paper can effectively improve the robot’s positioning accuracy.

### 4.2. Ablation Study Analysis

**Figure 13:**
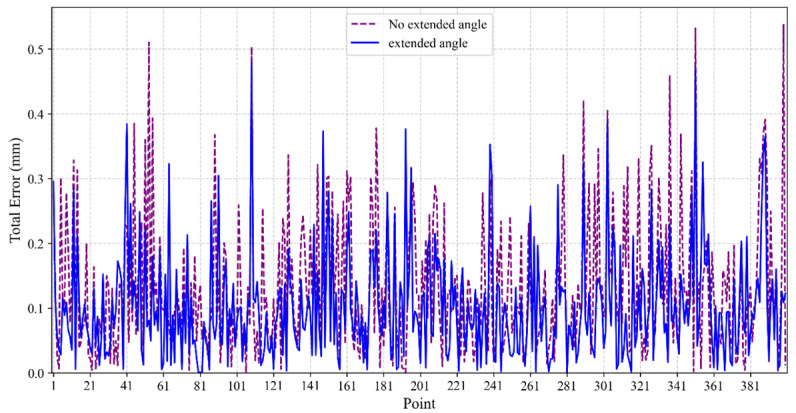
Comparison of total position errors using the model with extended angles (blue) versus without (purple).

To verify the improvement in the robot’s absolute positioning accuracy from the introduction of extended joint angles, a comparison was conducted. While keeping the model unchanged, one version used only the theoretical joint angles $\left[\theta_{1},\theta_{2},\cdots ,\theta_{6}\right]$ as the prediction model’s input. A comparison of its compensation results with the model that incorporates extended joint angles is shown in Figure 13. The prediction accuracies are presented in Table 3.

The results show that when the extended angles were introduced as features, the post-compensation maximum position error was reduced by 11.03%, the mean position error was reduced by 22.46%, and the standard deviation was reduced by 19.61%. This indicates that the introduction of the extended angles effectively reduces the error caused by gear backlash and improves the model’s positioning accuracy.

### 4.3. Comparative Experimental Analysis

**Figure 14:**
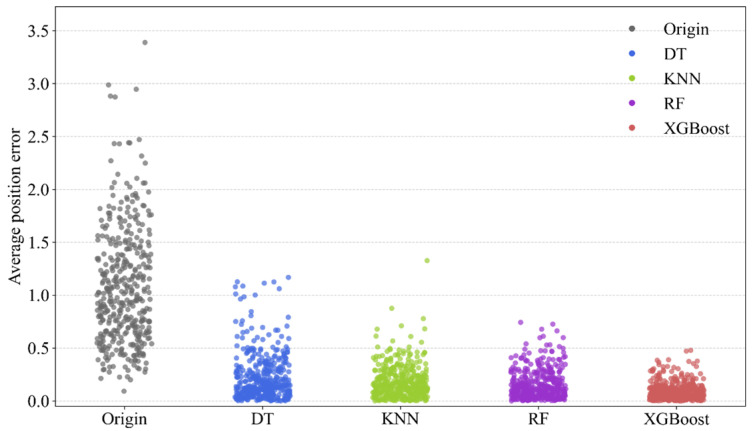
Swarm plot comparing the distribution of position errors for the original (uncompensated) data and after compensation by DT, KNN, RF, and XGBoost models.

**Figure 15:**
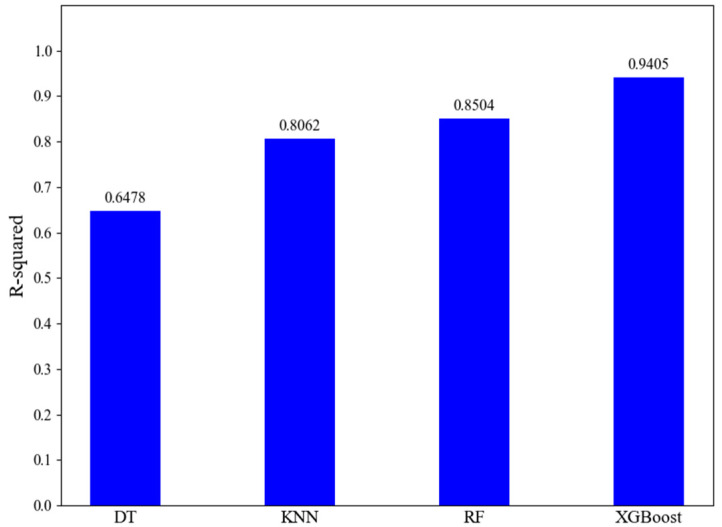
Comparison of the coefficient of determination (R-squared) values for the four machine learning models, indicating goodness-of-fit.

To validate the high prediction performance of the XGBoost model constructed in this study for handling robot spatial errors, a comparative experiment was conducted against the widely used Decision Tree (DT), K-Nearest Neighbors (KNN), and Random Forest (RF) models. The DT, KNN, and RF models each underwent 50 iterations of hyperparameter optimization using the Bayesian Optimization method. The optimal hyperparameter combinations were then used to construct the respective models, which were subsequently trained on the training set. The hyperparameter optimization process for all models followed the steps described in the preceding sections. The prediction accuracies of the final optimal XGBoost, KNN, and RF models on the test set are presented in the table. Figure 14 is provided showing a comparison of the position error compensation effects on the test set for the four methods. Figure 15 compares the goodness-of-fit for the four models.

Analysis of the experimental results from Figure 14 and Figure 15 and Table 4 indicates that the XGBoost-based robot spatial error prediction method is markedly effective for robot error pre-compensation. The XGBoost model’s effectiveness in reducing the mean position error showed an improvement of 55.42% over DT, 44.12% over KNN, and 34.80% over RF. For the reduction in the maximum position error, the XGBoost model demonstrated an improvement of 59.05% over DT, 63.95% over KNN, and 35.60% over RF. Therefore, the overall performance ranking of the four machine learning models for position error compensation is: XGBoost > RF > KNN > DT. This validates the effectiveness and balance of the BO-XGBoost-based robot spatial error prediction method proposed in this paper, which shows clear advantages in reducing robot position error compared to DT, KNN, and RF.

### 4.4. Validation on Continuous Trajectory

To evaluate the practical effectiveness of the proposed compensation method in a dynamic scenario, a continuous trajectory tracking experiment was conducted. A square path with dimensions of 180 mm × 180 mm was commanded in the robot’s workspace. The actual position of the robot’s end-effector was recorded, both with and without the compensation model activated.

**Figure 16:**
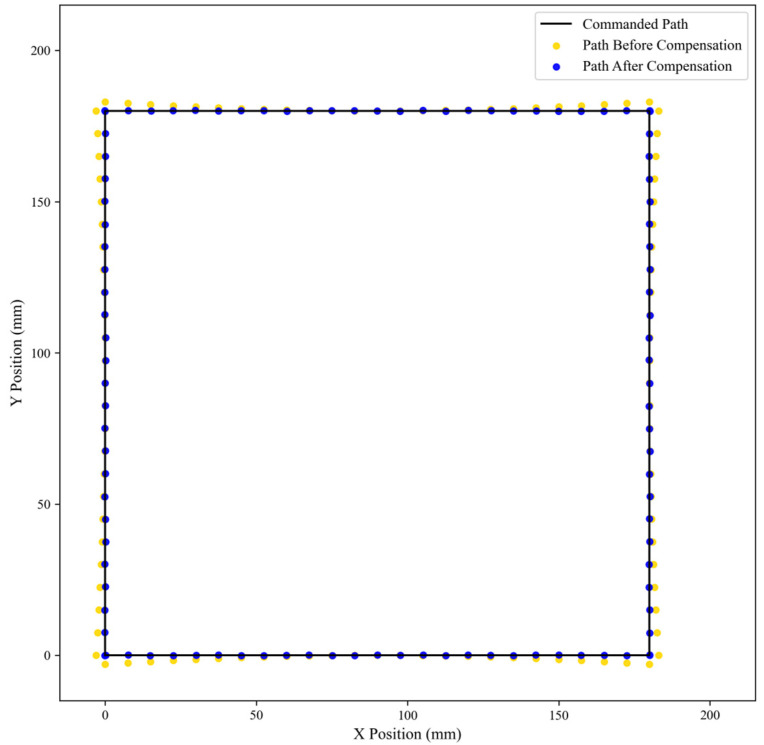
Comparison of commanded path versus actual trajectory with and without compensation for a square path.

The qualitative results of this validation are visualized in Figure 16. The commanded path is represented by a solid black line. The trajectory executed without compensation, depicted by yellow dots, exhibits significant and systematic deviations from the ideal path. On all four sides of the square, the uncompensated path consistently bows outwards, indicating a predictable error pattern during motion. In stark contrast, the trajectory executed with the proposed compensation model, shown as blue dots, aligns almost perfectly with the commanded path, visually confirming the model’s effectiveness.

To quantitatively assess this improvement, key trajectory error metrics were calculated, as presented in Table 5. The results show a dramatic reduction across all metrics, with the Mean absolute error (MAE) decreasing from 1.0833 mm to 0.1373 mm, an improvement of 87.32%. Similarly, the maximum deviation was reduced by 92.45%, from 3.0 mm down to just 0.2266 mm. While these residual dynamic errors (0.1373 mm) are slightly higher than those from the static point tests (0.1008 mm), this is expected due to additional factors in continuous motion such as controller tracking lag and vibrations.

Ultimately, these findings, combining both qualitative and quantitative evidence, strongly confirm the model’s powerful generalization capability. The model can successfully predict and correct errors during continuous motion, which is critical for practical applications such as welding, sealing, or machining.

### 4.5. Discussion

The effectiveness of the proposed method is substantiated by a comprehensive analysis of several key experimental results.

First, the ablation study in Section 4.2 directly validates the effectiveness of this study’s core innovation—the “extended joint angles”. By simply introducing the turning direction coefficient (λ), which reflects joint reversal information, the model’s mean position error was reduced by 22.46%, and the maximum error was reduced by 11.03%. This compelling result empirically confirms that joint reversal effects, such as gear backlash, are a significant contributor to the total absolute positioning error, a factor often overlooked in traditional compensation methods. This finding highlights a key advantage of data-driven approaches: the ability to effectively capture and compensate for complex physical phenomena without requiring complex first-principles modeling, thereby offering a more pragmatic and powerful pathway to high-accuracy robot calibration.

Second, superior features alone do not guarantee optimal performance; the choice of algorithm is equally critical. The comparative experiment in Section 4.3 clearly establishes the superiority of the BO-XGBoost model for this problem. When compared against other common machine learning models like Decision Tree (DT), K-Nearest Neighbors (KNN), and Random Forest (RF), XGBoost performed best across all key metrics. The overall performance ranking was: XGBoost > RF > KNN > DT. Specifically, the XGBoost model’s effectiveness in reducing the mean position error showed an improvement of 55.42% over DT, 44.12% over KNN, and 34.80% over RF. Furthermore, its R-squared value of 0.9405 was significantly higher than the other models, indicating that BO-XGBoost can more accurately fit the highly non-linear relationship between the robot’s joint configuration and its spatial positioning error.

Finally, the ultimate value of the model lies in its practical application. The continuous trajectory tracking experiment in Section 4.4 successfully extended the model’s validation from static point accuracy to a dynamic application scenario. The results clearly show that the uncompensated robot exhibited a significant, systematic outward bowing error when executing a square path. In contrast, with the proposed compensation model activated, the robot’s actual trajectory aligned almost perfectly with the commanded path, effectively eliminating the systematic bowing error and demonstrating the model’s powerful generalization capabilities.

In summary, this study captures key non-geometric error sources by introducing “extended joint angles”, constructs the most effective prediction model using a Bayesian-optimized XGBoost (Extreme Gradient Boosting) model, and verifies its high-fidelity performance in practical applications through dynamic trajectory experiments. This comprehensive solution provides an effective paradigm for enhancing the absolute positioning accuracy of industrial robots.

## 5. Conclusions

This paper proposed a spatial error prediction and compensation method for industrial robots using an XGBoost model optimized with Bayesian Optimization. By introducing a novel “extended joint angle” feature, the model effectively captured the complex, non-linear relationship between the robot’s configuration and its end-effector positioning error, including non-geometric effects like gear backlash.

The experimental results robustly demonstrated the method’s efficacy in two key scenarios. First, for discrete static points, the mean absolute position error was reduced from 1.0751 mm to 0.1008 mm, a 90.62% improvement. Second, a comparative analysis confirmed that the BO-XGBoost model outperformed traditional DT, KNN, and RF models, validating its suitability for high-precision applications. Furthermore, he model’s validity was confirmed in a dynamic scenario, where it also significantly reduced path deviation for a continuous square trajectory.

Similarly, this experiment also has limitations. which in turn suggest directions for future research. The experiments herein were conducted under a constant payload and at controlled speeds, and the model’s performance under variable operating conditions has not yet been investigated. Therefore, a primary focus of future work will be to examine the influence of varying payloads and operational speeds on positioning error, potentially by incorporating these parameters as additional input features. Furthermore, optimizing the algorithm for real-time implementation on robot controllers warrants further exploration to facilitate its transition from offline compensation to online, dynamic error correction.

## Figures and Tables

**Table 1:** Theoretical MD-H parameters of robot.

i	$\theta_{i}/rad$	$d_{i}/mm$	$a_{i}/mm$	$\alpha_{i}/rad$
1	0	0	0	0
2	0	−77.5	0	0
3	0	77.5	0	Π/2
4	−Π/2	−52.4	224	0
5	0	162	224	0
6	Π/2	80.5	0	−Π/2

**Table 2:** XGBoost hyperparameter performance table.

Hyperparameter	Search Range	Optimal Value	Number of Iterations
n_estimators	[200, 1500]	665	50
max_depth	[3, 12]	5	
learning_rate	[0.01, 0.15]	0.145	
subsample	[0.6, 1]	0.65	
reg_lambda	[1, 10]	7.6742	

**Table 3:** Comparative results of the ablation study on robot position error compensation.

Model	Average Error	Maximum Error	Standard Deviation of Error
No extended angle	0.1300	0.5375	0.1035
extended angle	0.1008	0.4782	0.0832

**Table 4:** Comparative experimental results for robot position error compensation.

Model	Average Error	Maximum Error	Standard Deviation of Error
Origin	1.0751	3.3884	0.5383
DT	0.2261	1.1678	0.2202
KNN	0.1804	1.3264	0.1543
RF	0.1546	0.7426	0.1381
XGBoost	0.1008	0.4782	0.0832

**Table 5:** Trajectory error metrics before and after compensation.

Error Metric	Before Compensation	After Compensation	Improvement/%
Mean absolute error	1.0833	0.1373	87.32
Root mean square error	1.4519	0.1437	90.10
Maximum deviation	3	0.2266	92.45

## Data Availability

The data presented in this study are available on request from the corresponding author.

## Abbreviations

The following abbreviations are used in this manuscript:

XGBoost	Extreme Gradient Boosting.
BO	Bayesian Optimization.
TCP	Tool Center Point.
ELM	Modified Denavit-Hartenberg.
MD-H	Modified Denavit-Hartenberg.
GBDT	Gradient Boosting Decision Tree.
DT	Decision Tree.
KNN	K-Nearest Neighbors.
RF	Random Forest.

## References

[1] Chen Y.B. (2017). Integrated and Intelligent Manufacturing: Perspectives and Enablers. Engineering.

[2] Zhu Z., Tang X., Chen C., Peng F., Yan R., Zhou L., Li Z., Wu J. (2022). High Precision and Efficiency Robotic Milling of Complex Parts: Challenges, Approaches and Trends. Chin. J. Aeronaut..

[3] Shen N., Guo Z., Li J., Tong L., Zhu K. (2018). A Practical Method of Improving Hole Position Accuracy in the Robotic Drilling Process. Int. J. Adv. Manuf. Technol..

[4] Dumas C., Caro S., Garnier S., Furet B. (2011). Joint Stiffness Identification of Six-Revolute Industrial Serial Robots. Robot. Comput. Integr. Manuf..

[5] Smith J., Doe A. (2019). A Review of Industrial Robot Accuracy and Repeatability. J. Manuf. Syst..

[6] Guo Y., Dong H., Wang G., Ke Y. (2016). Vibration Analysis and Suppression in Robotic Boring Process. Int. J. Mach. Tools Manuf..

[7] Wen X.L., Song A.G., Feng Y.G. (2022). Robot Calibration and Uncertainty Evaluation Based on Optimal Pose Set. Chin. J. Sci. Instrum..

[8] Chen X.Y., Zhang Q.J., Sun Y.L. (2019). Nonkinematic Calibration of Industrial Robots Using a Rigid-Flexible Coupling Error Model and a Full Pose Measurement Method. Robot. Comput.-Integr. Manuf..

[9] Nguyen H.N., Le P.N., Kang H.J. (2019). A New Calibration Method for Enhancing Robot Position Accuracy by Combining a Robot Model–Based Identification Approach and an Artificial Neural Network–Based Error Compensation Technique. Adv. Mech. Eng..

[10] Kim S.H. (2023). Feedforward Compensation of Contour Errors in Robotic Machining System Using Compliance Model. J. Manuf. Process..

[11] Zeng Y., Tian W., Li D., He X., Liao W. (2017). An Error Similarity-Based Robot Positional Accuracy Improvement Method for a Robotic Drilling and Riveting System. Int. J. Adv. Manuf. Technol..

[12] Fu Z., Pan J., Spyrakos-Papastavridis E., Lin Y.-H., Zhou X., Chen X., Dai J.S. (2020). A Lie-Theory-Based Dynamic Parameter Identification Methodology for Serial Manipulators. IEEE/ASME Trans. Mechatron..

[13] Yang J., Jin L., Han Z., Zhao D., Hu M. (2021). Analysis of Kinematic Parameter Identification Method Based on Genetic Algorithm. Proceedings of the Intelligent Robotics and Applications: 14th International Conference, ICIRA 2021.

[14] Schneider U., Drust M., Ansaloni M., Lehmann C., Pellicciari M., Leali F., Gunnink J.W., Verl A. (2016). Improving Robotic Machining Accuracy through Experimental Error Investigation and Modular Compensation. Int. J. Adv. Manuf. Technol..

[15] Chen D., Yuan P., Wang T., Ying C., Tang H. (2018). A Compensation Method Based on Error Similarity and Error Correlation to Enhance the Position Accuracy of an Aviation Drilling Robot. Meas. Sci. Technol..

[16] Aoyagi S., Kohama A., Nakata Y., Hayano Y., Suzuki M. Improvement of Robot Accuracy by Calibrating Kinematic Model Using a Laser Tracking System-Compensation of Non-Geometric Errors Using Neural Networks and Selection of Optimal Measuring Points Using Genetic Algorithm. Proceedings of the IEEE/RSJ International Conference on Intelligent Robots & Systems.

[17] Wang D., Bai Y., Zhao J. (2012). Robot Manipulator Calibration Using Neural Network and a Camera-Based Measurement System. Trans. Inst. Meas. Control..

[18] Angelidis A., Vosniakos G.C. (2014). Prediction and Compensation of Relative Position Error Along Industrial Robot Endeffector Paths. Int. J. Precis. Eng. Manuf..

[19] Li J., Heap D.A. (2014). Spatial Interpolation Methods Applied in the Environmental Sciences: A Review. Environ. Model. Softw..

[20] Zhang F., Shang W., Li G., Cong S. (2021). Calibration of Geometric Parameters and Error Compensation of Non-Geometric Parameters for Cable-Driven Parallel Robots. Mechatronics.

[21] Aalto J., Pirinen P., Heikkinen J., Venäläinen A. (2013). Spatial Interpolation of Monthly Climate Data for Finland: Comparing the Performance of Kriging and Generalized Additive Models. Theor. Appl. Climatol..

[22] Gan Y., Duan J., Dai X. (2019). A Calibration Method of Robot Kinematic Parameters by Drawstring Displacement Sensor. Int. J. Adv. Robot. Syst..

[23] Junde Q., Bing C., Dinghua Z. (2021). A Calibration Method for Enhancing Robot Accuracy through Integration of Kinematic Model and Spatial Interpolation Algorithm. Mech. Robot..

[24] Zeng Y., Tian W., Liao W. (2016). Positional Error Similarity Analysis for Error Compensation of Industrial Robots. Robot. Comput. Integr. Manuf..

[25] Lara-Molina F.A., Gonçalves R.S. (2024). Kinematic Reliability of Manipulators Based on an Interval Approach. Robotics.

[26] Bai Y. (2007). On the Comparison of Model-Based and Modeless Robotic Calibration Based on a Fuzzy Interpolation Method. Int. J. Adv. Manuf. Technol..

[27] Lightcap C., Hamner S., Schmitz T., Banks S. (2008). Improved Positioning Accuracy of the PA10-6CE Robot with Geometric and Flexibility Calibration. IEEE Trans. Robot..

[28] Bai M., Zhang M., Zhang H., Chen Z. (2021). Calibration Method Based on Models and Least-Squares Support Vector Regression Enhancing Robot Position Accuracy. IEEE Access.

[29] Cai Y., Yuan P., Shi Z., Chen D., Cao S. (2019). Application of Universal Kriging for Calibrating Offline-Programming Industrial Robots. J. Intell. Robot. Syst..

[30] Gao T., Meng F., Zhang X., Tian Z., Song H. (2023). An Operational Calibration Approach of Industrial Robots through a Motion Capture System and an Artificial Neural Network ELM. Int. J. Adv. Manuf. Technol..

[31] Min K., Ni F., Chen Z., Liu H., Lee C.-H. (2024). A Robot Positional Error Compensation Method Based on Improved Kriging Interpolation and Kronecker Products. IEEE Trans. Ind. Electron..

[32] Du Shanghai W., Yu T. Research on Positioning Error Compensation of Industrial Robot Based on BP Neural Network. Proceedings of the 2021 International Conference on Information Science, Parallel and Distributed Systems (ISPDS).

[33] Li Z., Li Y., Tian Y., Wang T. (2023). Research on Calibration and Error Compensation Method of an Industrial Robot Based on an Improved BP Neural Network. IEEE Trans. Instrum. Meas..

[34] Hu J.S., Hua F.F., Tian W. (2020). Robot Positioning Error Compensation Method Based on Deep Neural Network. J. Phys. Conf. Ser..

[35] Le P.-N., Kang H.-J. (2020). Robot Manipulator Calibration Using a Model Based Identification Technique and a Neural Network with the Teaching Learning-Based Optimization. IEEE Access.

[36] Zhang D., Shen S., Wu J., Wang F., Han X. (2023). Kinematic trajectory accuracy reliability analysis for industrial robots considering intercorrelations among multi-point positioning errors. Reliab. Eng. Syst. Saf..

[37] Craig J.J. (2005). Introduction to Robotics: Mechanics and Control.

[38] Cordes M., Brunke L., Hintze W. (2019). Correction to: Offline Simulation of Path Deviation Due to Joint Compliance and Hysteresis for Robot Machining. Int. J. Adv. Manuf. Technol..

[39] Ma L., Bazzoli P., Sammons P.M., Landers R.G., Bristow D.A. (2018). Modeling and Calibration of High-Order Joint-Dependent Kinematic Errors for Industrial Robots. Robot. Comput.-Integr. Manuf..

[40] He W., Zhang P., Guo K., Sun J., Sivalingam V., Huang X. (2023). Kinematic Calibration and Compensation of Industrial Robots Based on Extended Joint Space. IEEE Access.

[41] Chen T.Q., Guestrin C. XGBoost: A Scalable Tree Boosting System. Proceedings of the 22nd ACM SIGKDD International Conference on Knowledge Discovery and Data Mining (KDD 2016).

[42] Wang T., Bian Y., Zhang Y., Hou X. (2023). Classification of Earthquakes, Explosions and Mining-Induced Earthquakes Based on XGBoost Algorithm. Comput. Geosci..

[43] Geng X., Wu S., Zhang Y., Sun J., Cheng H., Zhang Z., Pu S. (2023). Developing Hybrid XGBoost Model Integrated with Entropy Weight and Bayesian Optimization for Predicting Tunnel Squeezing Intensity. Nat. Hazards.

[44] Zhang W., Wu C., Zhong H., Li Y., Wang L. (2021). Prediction of Undrained Shear Strength Using Extreme Gradient Boosting and Random Forest Based on Bayesian Optimization. Geosci. Front..

[45] Panteleev A.V., Lobanov A.V. (2020). Mini-Batch Adaptive Random Search Method for the Parametric Identification of Dynamic Systems. Autom. Remote Control..

[46] Bhat P.C., Prosper H.B., Sekmen S., Stewart C. (2018). Optimizing Event Selection with the Random Grid Search. Comput. Phys. Commun..

[47] Ghahramani Z. (2015). Probabilistic Machine Learning and Artificial Intelligence. Nature.

[48] Xia Y., Liu C., Li Y., Liu N. (2017). A Boosted Decision Tree Approach Using Bayesian Hyper-Parameter Optimization for Credit Scoring. Expert Syst. Appl..

